# Theoretical and experimental study of attenuation in cancellous bone

**DOI:** 10.1117/1.JBO.29.S1.S11526

**Published:** 2024-03-19

**Authors:** Wenyi Xu, Weiya Xie, Dong Yu, Haohan Sun, Ying Gu, Xingliang Tao, Menglu Qian, Liming Cheng, Hao Wang, Qian Cheng

**Affiliations:** a Tongji University, Institute of Acoustics, School of Physics Science and Engineering, Shanghai, China; b Tongji University, Key Laboratory of Spine and Spinal Cord Injury Repair and Regeneration of Ministry of Education, Shanghai, China; c National Key Laboratory of Autonomous Intelligent Unmanned Systems, Shanghai, China; d Frontiers Science Center for Intelligent Autonomous Systems, Ministry of Education, Shanghai, China

**Keywords:** cancellous bone, high-frequency viscous corrections, Biot’s theory, photoacoustic differential attenuation spectrum, acoustic propagation characteristic, osteoporosis

## Abstract

**Significance:**

Photoacoustic (PA) technology shows great potential for bone assessment. However, the PA signals in cancellous bone are complex due to its complex composition and porous structure, making such signals challenging to apply directly in bone analysis.

**Aim:**

We introduce a photoacoustic differential attenuation spectrum (PA-DAS) method to separate the contribution of the acoustic propagation path to the PA signal from that of the source, and theoretically and experimentally investigate the propagation attenuation characteristics of cancellous bone.

**Approach:**

We modified Biot’s theory by accounting for the high frequency and viscosity. In parallel with the rabbit osteoporosis model, we build an experimental PA-DAS system featuring an eccentric excitation differential detection mechanism. Moreover, we extract a PA-DAS quantization parameter—slope—to quantify the attenuation of high- and low-frequency components.

**Results:**

The results show that the porosity of cancellous bone can be evaluated by fast longitude wave attenuation at different frequencies and the PA-DAS slope of the osteoporotic group is significantly lower compared with the normal group (**p<0.01).

**Conclusions:**

Findings demonstrate that PA-DAS effectively differentiates osteoporotic bone from healthy bone, facilitating quantitative assessment of bone mineral density, and osteoporosis diagnosis.

## 1 Introduction

Osteoporosis and fractures stemming from it have emerged as chronic diseases adversely affecting the health of the elderly.^1^ As populations age, the annual global increase in the number of fracture patients is ∼8.9  million, leading to a considerable rise in public health costs.^2^^–^^4^ Therefore, early diagnosis of osteoporosis not only can prevent fractures but also substantially reduce healthcare expenditures and resource usage.

Compared to dense bone, the microstructure and chemical composition of cancellous bone are more sensitive to osteoporosis, as evidenced by reduced trabecular thickness, connectivity, and number, along with increased lipid content,^5^^–^^8^ suggesting that cancellous bone is ideally suited as a diagnostic site for early osteoporosis. However, the unique structural characteristics of cancellous bone make its detection and quantitative evaluation challenging, thereby complicating the early diagnosis of osteoporosis. The primary detection methods employed in clinical and research settings for osteoporosis include dual-energy X-ray absorptiometry (DEXA), quantitative computed tomography (QCT), magnetic resonance imaging (MRI), and quantitative ultrasound (QUS).^9^^–^^12^ DEXA serves as the “gold standard,” primarily because it provides the best predictor of osteoporotic fractures through bone mineral density (BMD) information.^13^ However, DEXA accounts for only 60% to 70% of changes in bone strength and lacks information on microstructure and elasticity.^14^ QCT extends bone analysis from two to three dimensions, providing both volumetric BMD and microstructural characteristics.^15^^,^^16^ However, its utility is hampered by radiation hazards and a lack of chemical information. MRI can identify alterations in bone marrow fat content and microstructure, facilitating early diagnosis.^17^^–^^19^ Nonetheless, its high cost and operational complexity limit its widespread use.^20^^,^^21^ QUS, being radiation-free, quick, affordable, and user-friendly, has gained traction as a powerful tool for screening bone quality.^5^^,^^22^^,^^23^ Initially centered on cortical bone,^24^^–^^26^ QUS is increasingly being used to explore cancellous bone characteristics due to its heightened sensitivity to osteoporotic changes. It provides physical information on BMD, bone microstructure, and mechanical properties, mainly by detecting the speed of sound (SoS) and broadband ultrasound (US) attenuation.^12^^,^^27^ However, it falls short of detecting changes in the organic chemical composition of bone tissue.

Photoacoustic (PA) techniques offer both chemical and physical insights into biological tissues and have been utilized for tissue identification and disease detection, including osteoporosis.^28^^–^^32^ During the past decade, considerable advancements have been made in PA-based bone evaluation. Mandelis and Lashkari^33^ established a set of photoacoustic-ultrasound (PA-US) backscattering detection systems, successfully detecting minute changes in the BMDs of both cancellous and cortical bones. Their study indicated that the apparent integral backscattering coefficient decreases as the collagen content decreases.^34^^–^^36^ Wang^37^^,^^38^ employed three-dimensional PA imaging (PAI) and power spectral analysis to achieve both qualitative and quantitative evaluations of bone microstructure. He also introduced multispectral PA decoupling and thermal PA methods for quantitative evaluation of the organic and inorganic chemical components in cancellous bone.^37^^–^^39^ Additionally, a combined PA-US system was developed, verifying the *in vivo* feasibility of assessing human calcaneus microstructure and chemical composition.^40^ Steinberg developed a multispectral PA-US dual-mode system capable of *in vivo* detection of fat and blood ratios in tibial bone marrow.^41^ The SoS for the first arriving wave in the tibia showed a strong correlation with the SoS value based on QUS.^42^ Feng et al.^43^ simulated various aspects of PA in skeletal tissues, including light attenuation and distribution, along with PA signal generation and propagation, based on a three-dimensional model. They proposed a PA physicochemical spectral method for assessing changes in the chemical composition and microstructure of cancellous bone.^44^^,^^45^ Our group has leveraged the MWPA time-frequency spectral analysis method to evaluate both the chemical content (minerals and lipids) and microstructure of bone tissues, based on the distinct optical absorption characteristics and sizes of various chromophores present.^46^^,^^47^ The preceding research in this field demonstrates the successful application of various PAI and spectral analysis methods for osteoporosis diagnosis, making it possible to assess BMD, bone microstructure, and chemical composition in a comprehensive manner.^48^

Nevertheless, the intricacy of PA signals, which arise from the two-phase, solid–liquid porous structure of cancellous bone, presents a significant challenge in using PA techniques for imaging of bone marrow distribution in cancellous bone.^49^ First, PA signals generated from various chemical clusters within cancellous bone with different microscales have complex optical and ultrasonic spectral distributions. In addition, these chemical clusters have uneven spatial distributions, adding another layer of complexity to the spatial distribution of PA sources. Thus the PA sources of cancellous bone possess a threefold complexity. Second, as these PA signals navigate through the cancellous bone, they undergo multiple scattering and attenuation events. Therefore, the final PA signal received by the transducers is a composite of broadband signals from distributed PA sources and the acoustic propagation characteristics specific to the porous cancellous bone. Decoupling these signals could yield valuable multidimensional insights into cancellous bone.

In this paper, we do a focused study—both theoretical and experimental—on the high-frequency broadband PA signal propagation generated by bone marrow in cancellous bone. Theoretically, we apply high-frequency and viscous corrections to Biot’s theory, tailoring it for bone marrow PA signal propagation in two-phase solid–liquid porous media. The numerical simulations of PA wave modes and velocity and attenuation coefficients are also employed for validation. In the method, we propose a PA differential attenuation spectrum (PA-DAS) method to remove the contribution of PA sources on PA signals in the frequency domain. Subsequently, *ex vivo* experiments are performed to measure PA-DAS, and a quantitative PA-DAS parameter is extracted to evaluate BMD and diagnose osteoporosis. Our results highlight the potential utility of this method for comprehensive bone quality assessment and lay a foundation for the further PAI study of bone marrow distribution in cancellous bone.

## 2 Modified Biot’s Theory

Cancellous bone is a complex porous medium composed of solid trabecular bone interspersed with fluid-filled bone marrow clusters, making it a typical example of a porous, elastic, viscous medium. Biot and Willis^50^^–^^55^ established the elastic theory for understanding acoustic propagation in such saturated solid–liquid two-phase porous media, thereby enabling further theoretical research in the field. Biot’s theory has subsequently found applications in the nondestructive evaluation of bone using biomedical US.^56^^–^^62^ However, Biot’s theory is limited to cases where the acoustic frequency is below the medium’s critical frequency $f_{c}$, and the flow of liquid through the pores is well-described by Poiseuille flow. The critical frequency $f_{c}$ is defined as^63^

$$f_{c}=\frac{\phi \eta }{2\pi \rho_{f}K},$$ (1)

where ϕ denotes the porosity, η denotes the fluid viscosity, $\rho_{f}$ represents the fluid density, and K denotes the permeability. For cancellous bone filled with viscous bone marrow, $f_{c}$ is typically in the range of 1 to 10 kHz.^64^^,^^65^ The sizes of the trabecular bone vary from 50 to 200  μm, while the trabecular spaces (bone marrow clusters) range from 0.2 to 3 mm.^66^ The SoS in trabecular bone and bone marrow are 3200 and 1500  m/s, respectively.^67^ Notably, the frequencies of PA signals generated in these structures exceed 220 kHz,^68^ far surpassing $f_{c}$, as we discussed in Appendix A2 in Supplementary Material. This means that the laminar flow condition described by Poiseuille’s law no longer holds, necessitating modifications to Biot’s theory for high-frequency applications.

In addition, the viscous nature of the fluid bone marrow leads to energy dissipation due to the relative motion between the fluid and the solid trabecular framework, further indicating the need for viscosity corrections. To account for the dissipation of acoustic wave propagation in a solid–liquid two-phase porous medium, the governing equations of motion can be expressed as follows:^54^^,^^55^

$$\mu_{b}\nabla^{2}\overset{⇀}{u}+(\lambda_{b}+\mu_{b})\nabla e+Q\nabla \epsilon =\frac{\partial^{2}}{\partial t^{2}}(\rho_{11}\overset{⇀}{u}+\rho_{12}\overset{⇀}{U})+b\frac{\partial }{\partial t}(\overset{⇀}{u}-\overset{⇀}{U}),$$ (2)

$$Q\nabla e+R\nabla \epsilon =\frac{\partial^{2}}{\partial t^{2}}(\rho_{12}\overset{⇀}{u}+\rho_{22}\overset{⇀}{U})-b\frac{\partial }{\partial t}(\overset{⇀}{u}-\overset{⇀}{U}).$$ (3)

where $\vec{u}$ denotes the solid skeleton displacement vector, and $\vec{U}$ represents the liquid displacement vector. The elastic constants $\mu_{b}$ and $\lambda_{b}$ characterize the frame’s material elasticity and also depend on structural parameters, such as porosity ϕ. R and Q are the additional elastic constants, with Q being Biot’s constant that describes the coupling between the liquid and solid phases. $e=e_{11}+e_{22}+e_{33}=\nabla \cdot \overset{⇀}{u}$ denotes the normal strains for the solid, $\epsilon =\epsilon_{11}+\epsilon_{22}+\epsilon_{33}=\nabla \cdot \overset{⇀}{U}$ denotes the strain for fluid, whereas $\rho_{mn}$ are the mass coefficients related to the porosity ϕ and the mass densities $\rho_{s}$ and $\rho_{f}$ of the solid, and fluid $\rho_{12}$ is the mass coupling. $\rho_{12}$ is related to dynamic tortuosity, $\overset{¯}{\rho_{12}}=\phi \cdot \rho_{f}[1-\alpha_{\infty }]-\frac{Z}{\sqrt{i\cdot \omega }}$, where $Z=\frac{2\cdot \phi }{\Lambda }\sqrt{\rho_{f}\cdot \eta }$, Λ is the viscous characteristic length, and $\alpha_{\infty }$ is the dynamic tortuosity.^69^ For a variety of smooth pore shapes, the relationship between viscous characteristic length Λ and the pore form factor M can be represented by $M=\frac{8\alpha_{\infty }k_{0}}{\Lambda^{2}\phi }$.^66^ The parameter $b=\frac{\eta \phi^{2}}{K}$ serves as a dissipation factor and is a function of the porosity ϕ.

The Fourier transform solution of velocities for the shear wave ($c_{T}^{*}$) and longitude wave ($c_{L1}^{*}$ and $c_{L2}^{*}$) in solid–liquid two-phase porous media with viscous losses can be expressed as follows:^70^

$$c_{T}^{*2}=N(\rho_{22}+\frac{b}{i\omega })/[(\rho_{11}+\frac{b}{i\omega })(\rho_{22}+\frac{b}{i\omega })-{\left(\rho_{12}-\frac{b}{i\omega }\right)}^{2}]\;=N\rho_{22}(\omega )/[\rho_{11}(\omega )\rho_{22}(\omega )-\rho_{12}^{2}(\omega )],$$ (4)

$$c_{\text{L}j}^{*2}=\frac{(M+\frac{b}{i\omega }H)\pm \sqrt{{\left(M+\frac{b}{i\omega }H\right)}^{2}-4L(\rho_{11}\rho_{22}-\rho_{12}^{2}+\frac{b}{i\omega }\rho )}}{2(\rho_{11}\rho_{22}-\rho_{12}^{2}+\frac{b}{i\omega }\rho )},\;(j=1,2),$$ (5)

where the elastic coefficient $N=\mu_{\text{b}}$, $\rho_{11}(\omega )=\rho_{11}+\frac{b}{i\omega }$, $\rho_{12}(\omega )=\rho_{12}-\frac{b}{i\omega }$, $\rho_{22}(\omega )=\rho_{22}+\frac{b}{i\omega }$, H=(P+R+2Q), $L=(PR-Q^{2})$, $M=(R\rho_{11}+P\rho_{22}-2Q\rho_{12})$, $\rho =\rho_{11}+\rho_{22}+2\rho_{12}$, and ω denotes the angular frequency.

The velocity of shear wave ($c_{\text{T}}$) and two types of longitude wave ($c_{\text{L}1}$ and $c_{\text{L}2}$) in solid–liquid two-phase porous media without viscosity is given by^54^^,^^55^

$$c_{\text{T}}^{2}=N\rho_{22}/(\rho_{11}\rho_{22}-\rho_{12}^{2}),$$ (6)

$$c_{\text{L}j}^{2}=\frac{M\pm \sqrt{M^{2}-4L(\rho_{11}\rho_{22}-\rho_{12}^{2})}}{2(\rho_{11}\rho_{22}-\rho_{12}^{2})},\;(j=1,2).$$ (7)

By comparing the velocities in solid–liquid two-phase porous media with and without viscosity, we observe that the viscosity loss alters Biot’s mass coefficients $\rho_{mn}$ into complex quantities. This results in the shear wave velocity and yields the fast and slow longitudinal wave velocities that are complex numbers. As sound waves propagate through a dissipative solid–liquid two-phase porous medium, their amplitudes decay progressively with increased propagation distance.

To account for high-frequency dissipation, Biot’s mass coefficients can be transformed as follows:

$$\rho_{mn}(\omega )=\rho_{mn}+(-1{)}^{m+n}\frac{bF(\kappa )}{i\omega },\;(m,n=1,2).$$ (8)

where $F(\kappa )=\frac{1}{3}\frac{i^{1/2}\kappa \;\tanh(i^{1/2}\kappa )}{1-\frac{1}{i^{1/2}\kappa }\;\tanh(i^{1/2}\kappa )}$ is a complex number that represents the deviation from Poiseuille’s law as frequency increases, reflecting the phase difference between velocity and friction forces.^54^ The frequency-dependent dissipation coefficient $\kappa =a(\frac{2\pi f\rho_{f}}{\eta }{)}^{\frac{1}{2}}$, where a is the average pore size in the porous medium.

The attenuation coefficients are determined by the imaginary parts of the complex wave numbers:

αT*=−Im(k)=−Im(2πfcT*), (9)

αLj*=−Im(k)=−Im(2πfcLj*),(j=1,2). (10)

The high-frequency dissipation coefficient $\alpha^{*}$ is influenced by several factors, including the porosity ϕ, the average pore size a of the solid–liquid two-phase porous medium, the frequency f of the acoustic wave, and the fluid viscosity η. Therefore, the porosity ϕ of cancellous bone can be inferred based on the frequency-dependent attenuation coefficient.

## 3 Numerical Simulations

### 3.1 Numerical Simulation Parameters

Based on the above modified Biot’s theory, we numerically simulated the propagation of acoustic waves in porous cancellous bone using MATLAB (version R2020b, MathWorks, Natick, Massachusetts, United States). The parameters used for these simulations are listed in Tables 1 and 2. It should be noted that, in general, the porosity of normal cancellous bone is ∼0.73,^73^^,^^74^ and that of osteoporotic cancellous is larger than this value. Therefore, we mainly conducted research using the porosity range of 0.72 to 0.90. We specifically examined the influences of porosity and sound frequency on sound velocity and attenuation.

**Table 1:** Structural and acoustic parameters of cancellous bone.^66^

Parameters	Value
Young’s modulus of solid bone $E_{s}$	22 GPa
Poisson’s ratio of solid bone $v_{s}$	0.32
Poisson’s ratio of skeletal frame $v_{\text{b}}$	0.32
Compressibility modulus of the solid $\kappa_{s}$	20.37 GPa
Solid density $\rho_{s}$	$1960\;\mathrm{kg}/m^{3}$
Fluid density $\rho_{f}$	$1000\;\mathrm{kg}/m^{3}$
Compressibility modulus of the solid $\kappa_{f}$	2.28 GPa
Fluid viscosity $\eta_{f}$	0.001 Pa·s
Power index n	$n(\theta )=1.43\;\cos^{2}(\theta )+2.14\;\sin^{2}(\theta )$
Tortuosity $\alpha_{\infty }$	$\alpha_{\infty }=1-0.259(1-\frac{1}{\phi })+0.864\;\cos^{2}(\theta )$
Pore size a	1 mm
Form factor M	4

**Table 2:** Permeability of different porosities.^71^^,^^72^

Porosity ϕ	Permeability $k_{0}$ ($m^{2}$)
0.72	$5\times 10^{-9}$
0.75	$7\times 10^{-9}$
0.80	$2\times 10^{-8}$
0.83	$3\times 10^{-8}$
0.90	$8\times 10^{-8}$

### 3.2 Numerical Simulation Results

**Fig. 1:**
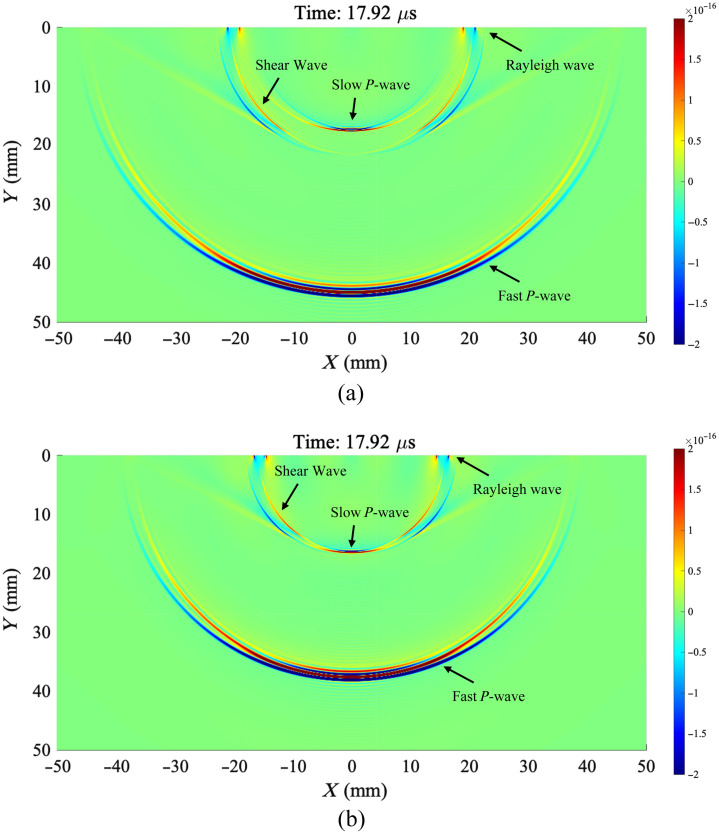
Numerical simulation results of the sound field of PA wave generated by photoexcited bone marrow clusters on the boundary of semi-infinite cancellous bone propagating in cancellous bone with porosities of (a) 0.72 and (b) 0.83 at 17.92  μs (Video 1, mp4, 3.165 MB [URL: https://doi.org/10.1117/1.JBO.29.S1.S11526.s1]; Video 2, mp4, 3.094 MB [URL: https://doi.org/10.1117/1.JBO.29.S1.S11526.s2]).

Based on the modified Biot’s theory outlined above, we establish a computational model of semi-infinite cancellous bone and numerically simulate the propagation of PA waves in cancellous bone generated by bone marrow on the bone surface irradiated by a pulsed spotlight with a diameter of 2 mm. Figures 1(a) and 1(b) demonstrate the PA field at 17.92  μs in the cancellous bone with 0.72 and 0.83 porosity, respectively, including fast longitude wave (fast P wave), slow longitude wave (slow P wave), shear wave, and Rayleigh waves (for the complete 0 to 17.92  μs sound field simulation, see Video 1 for a porosity of 0.72 and Video 2 for a porosity of 0.83). The numerical simulation shows that the speed and amplitude of fast P waves are much larger than those of other wave modes and other wave modes, which means that we can extract very clean fast P waves from the PA signal in the time domain.

**Fig. 2:**
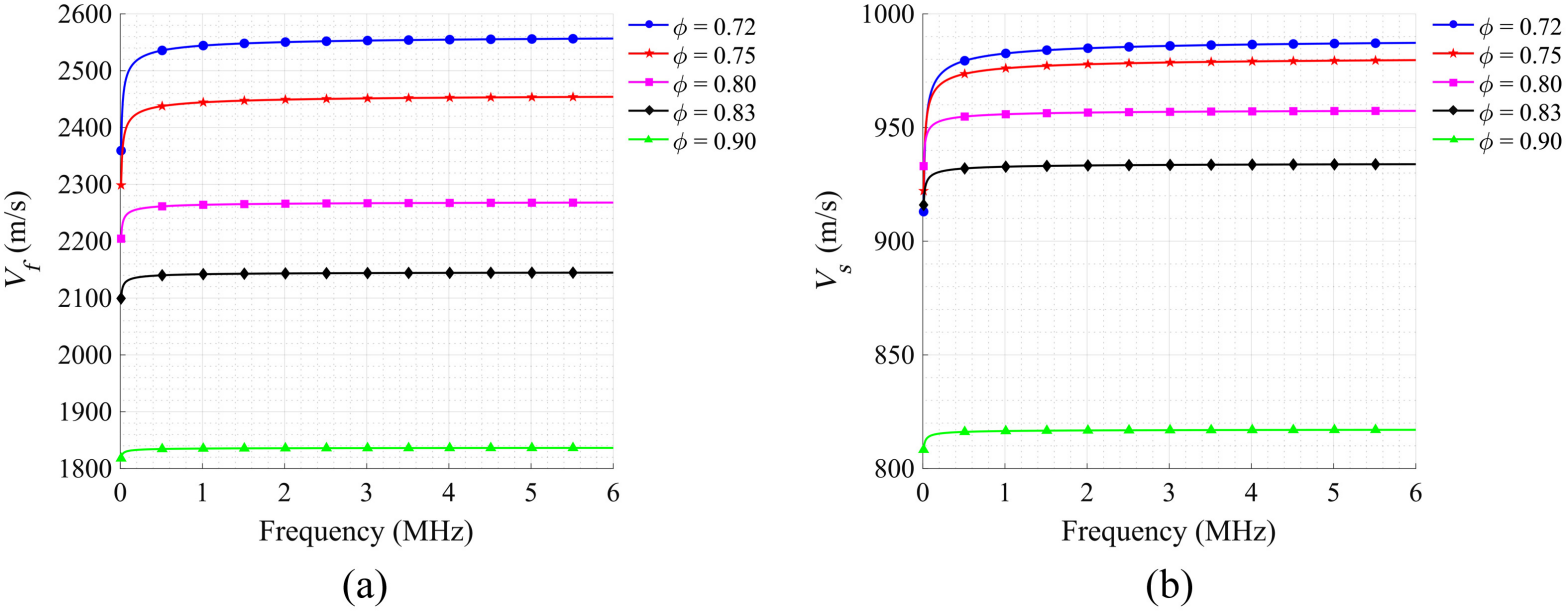
Numerical simulation results. SoS of (a) fast and (b) slow longitude waves as a function of frequency at different porosities.

Figures 2(a) and 2(b) illustrate the calculated trend of the velocity of fast and slow P waves propagating in solid–liquid two-phase porous media with porosity and frequency, respectively. Similarly, in the calculated porosity range of 0.72 to 0.90, the velocity of fast P wave ($c_{\text{f}}$) is much higher than that of slow P wave ($c_{s}$) at the same porosity. Notably, both fast and slow P wave velocities are insensitive to frequency change, that is, there is almost no dispersion, which is very advantageous for PA detection because the bandwidth of the PA signal is usually broad. However, both velocities decrease significantly with increasing porosity, which is also consistent with Fig. 1. This is mainly because the fact that as the porosity increases, the proportion of the solid phase decreases and the solid–liquid coupling becomes weaker, leading to a decrease in the velocity of the fast and slow waves, respectively.

**Fig. 3:**
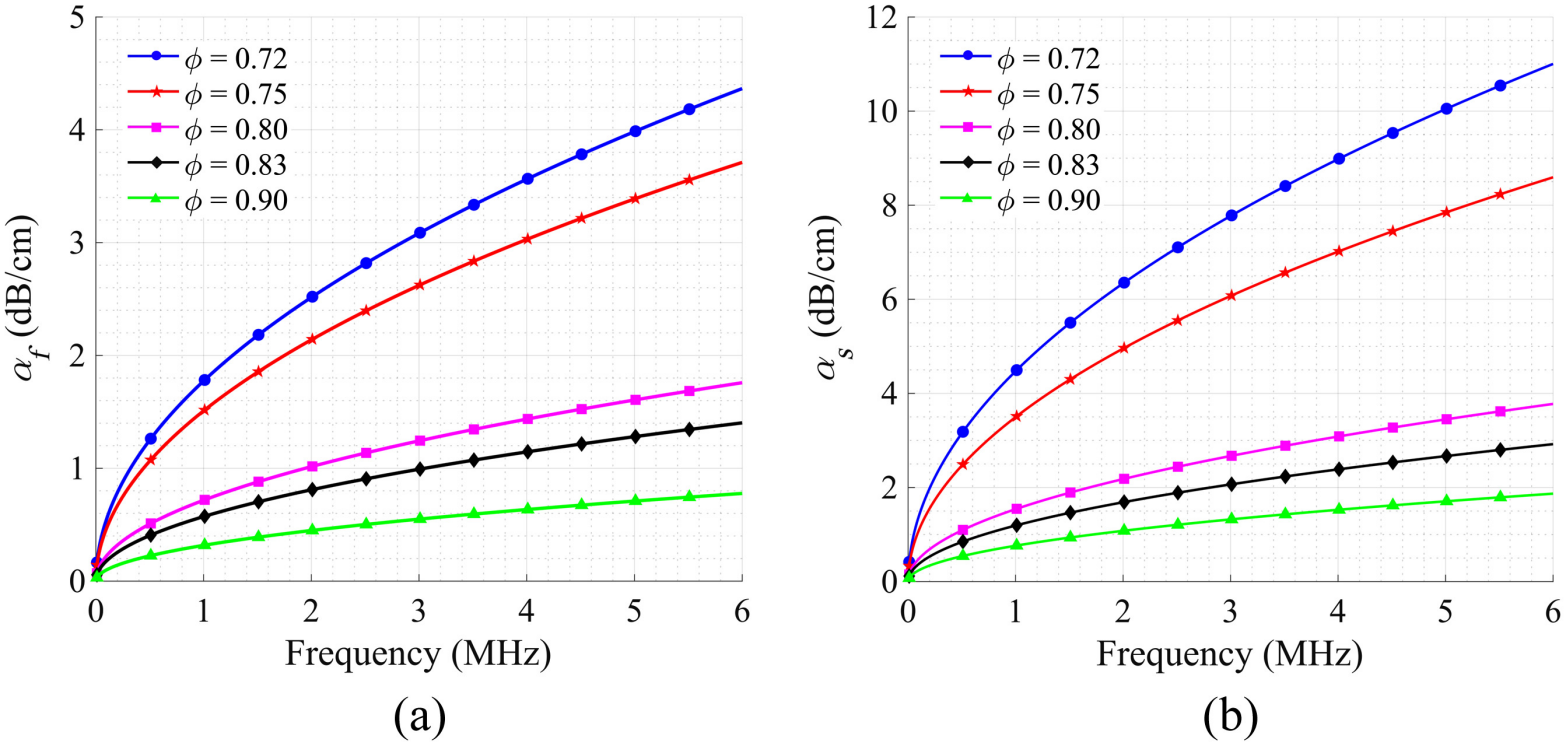
Numerical simulation results. Viscous absorption attenuation coefficients of (a) fast and (b) slow longitude waves as a function of frequency at different porosities.

Figures 3(a) and 3(b) showcase the viscous absorption attenuation coefficients for fast and slow P waves as they travel through solid–liquid two-phase porous media, respectively. In the calculated porosity range of 0.72 to 0.90, the absorption attenuation coefficient for the fast P wave is much smaller than that of the slow P wave with the same porosity. In addition, these attenuation coefficients are influenced by both the porosity of the cancellous bone and the frequency of the acoustic waves. When the porosity is constant, the absorption attenuation coefficients for both fast and slow waves increase with increasing frequency. The attenuation coefficients exhibit a shift from fast to slow changes, eventually tending toward a linear pattern within the frequency range of 1 to 6 MHz as the frequency increases. Conversely, when the frequency is held constant, the absorption attenuation coefficient decreases as the porosity increases. This is mainly because as the porosity of cancellous bone increases, the solid–liquid interface area decreases, which in turn leads to a reduction in energy dissipation caused by the relative motion between the solid bone trabecular frame and the liquid bone marrow.

These simulation results indicate that the fast P wave with high speed and low attenuation is more suitable for the PA detection of cancellous bone, and the porosity ϕ of porous media can be evaluated based on the attenuation of acoustic waves across various frequencies.

## 4 PA-DAS Method

As discussed earlier, the PA signal arriving at the transducers is a complex mixture of broadband signals originating from distributed PA sources and the inherent acoustic propagation characteristics of porous cancellous bone. The relationship of PA signals received by the transducers, $p_{1}(t_{1})$ and $p_{2}(t_{2})$, in the time domain, can be represented as

$$p_{2}(t_{2})=p_{1}(t_{1})⊗h(t_{2}-t_{1}),$$ (11)

where ⊗ denotes the convolution operator, $p_{1}(t_{1})$ represents the PA signal generated by bone tissue at time $t_{1}$, and $p_{2}(t_{2})$ represents the PA signal after propagation of the cancellous bone. And $t_{1}$ is any time point in $p_{1}$, $t_{2}$ is the corresponding time point of $t_{1}$ in $p_{2}$ after propagation, $t_{2}-t_{1}$ represents the propagation time of the signal in the system. h(t) denotes the system impulse response of bone tissue. Applying the Fourier transform to Eq. (11) yields the spectrum of PA signals arriving at the transducers:

$$P_{2}(\omega )=P_{1}(\omega )H(\omega )e^{j\omega (t_{2}-t_{1})},$$ (12)

where $P_{1}(\omega )$ and $P_{2}(\omega )$ denote the Fourier transforms of $p_{1}(t_{1})$ and $p_{2}(t_{2})$, respectively, and H(ω) is the Fourier transform of $h(t_{2}-t_{1})$. We can derive the frequency-related acoustic propagation characteristics of cancellous bone by calculating the differential attenuation spectrum of the signal after propagation and the PA signal generated by sources:

$$H(\omega )=\frac{\left|P_{2}(\omega )\right|}{\left|P_{1}(\omega )\right|}.$$ (13)

Figure 3 shows that the attenuation coefficient is approximately linear within the 1 to 6 MHz range. Thus the acoustic propagation characteristics within this frequency band can be linearly fitted to quantify attenuation across different frequencies.

## 5 *Ex vivo* Experiments on Rabbit Bone Specimens

Based on the results of theoretical and numerical simulations, the PA experiment was conducted on a rabbit osteoporosis model to verify the feasibility of bone evaluation based on PA-DAS.

### 5.1 Animal Model and Bone Tissue Sample

**Fig. 4:**
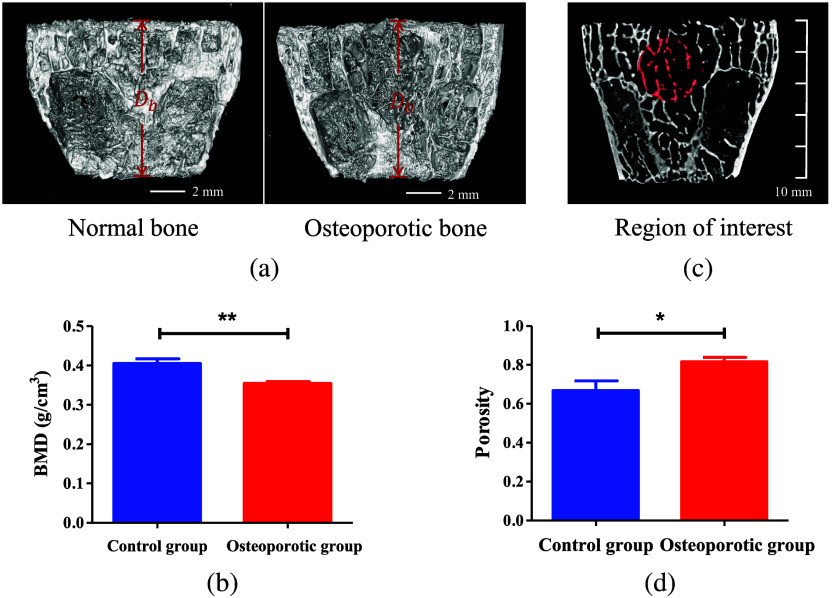
Micro-CT results: (a) 3D images of normal bone and osteoporotic bone. (b) Statistical analysis results of BMD of bone specimens from the osteoporotic group (n=6) and control group (n=5) (**p<0.01). (c) ROI used to calculate porosity. (d) Statistical analysis results of porosity of bone specimens from the osteoporotic groups (n=6) and control group (n=5) (*p<0.05).

In our study, we used a sample of eleven 5-month-old female New Zealand white rabbits. Six were randomly selected to undergo ovariectomy, forming the experimental group, while the remaining five received a sham operation to serve as the control group. After 5 months of identical living conditions, all rabbits were euthanized. The left metaphyseal region was then carefully dissected and sectioned into slices with a uniform thickness of 1.5 mm. Dense peripheral bone tissue was removed, and the slices were further trimmed to a standard width $D_{b}$ equal to 10 mm, as shown in Fig. 4(a), making them suitable for the PA experiments.

### 5.2 Gold Standard-BMD

Following the PA experiments, all eleven bone samples from both the experimental and control groups were subjected to microcomputed tomography scanning (Micro-CT, venus001, Avatar3, Pingsheng Life Medical Technology, Shanghai, China). Three-dimensional images of both the osteoporotic and normal bones are presented in Fig. 4(a). As evidenced by Fig. 4(b), the BMD of the normal bones in the control group was significantly higher than that of the osteoporotic bones in the experimental group (p<0.01). In Fig. 4(d), the statistical analysis of the region of interest (ROI) from Fig. 4(c) reveals a significantly higher porosity in the osteoporotic group compared with the control group (p<0.05). Literature suggests that higher BMD is inversely related to porosity.^73^^–^^77^ Therefore, it can be concluded that osteoporotic bone exhibits higher porosity compared with normal bone.

### 5.3 PA Experimental Setup

**Fig. 5:**
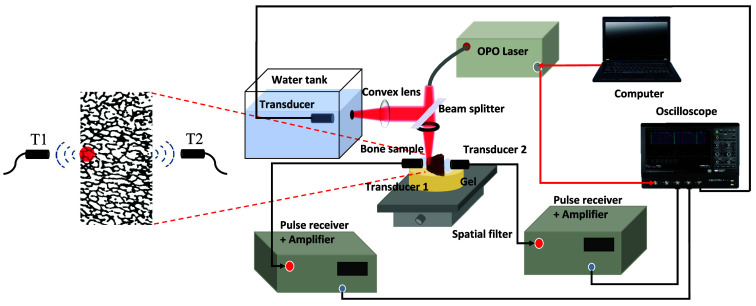
Schematic of experimental setup for PA measurements of bone samples.

To measure the frequency-dependent attenuation of PA signals in cancellous bone, we propose an eccentric excitation differential detection system specifically for PA experiments. Figure 5 illustrates the schematic layout of the experimental setup. A tunable optical parametric oscillator laser (Phocus Mobile, OPOTEK, Carlsbad, California, United States) produces laser pulses with durations ranging from 2 to 5 ns. We selected a laser wavelength of 1730 nm, which corresponds to the significant specific absorption wavelength of lipids^78^^,^^79^—a major component of bone marrow clusters^80^—to irradiate the bone samples and thereby excite PA signals.^81^^,^^82^ To compensate for variations in laser energy over time, a spectrophotometer with a 9:1 transmittance-to-reflectance ratio was used to split the laser beam into two paths. One path was focused using a convex lens to irradiate a blackbody, and the other was weakly focused on one side of the bone tissue sample surface, tangent to the side of the sample, forming a light spot with a diameter $D_{l}$ of ∼2  mm. The bone sample was placed on a 5 cm thick phantom to mitigate any direct light or sound reflections from the platform. As shown in Fig. 5, a needle hydrophone T1 (HNC1500, ONDA Corporation, Sunnyvale, California, United States) with a bandwidth of 0 to 20 MHz was placed on the side of the sample near the light spot to receive the unattenuated PA signals. These signals were then amplified by 25 dB using an amplifier (5072PR, Olympus Corporation, Tokyo, Japan) and subjected to 1 MHz high-pass filtering to remove low-frequency noise. On the opposite side of the light source, a planar transducer with a center frequency of 2.25 MHz (Olympus Corporation, Tokyo, Japan) was positioned to receive the PA signals transmitted through the cancellous bone. These signals were amplified by another 25 dB using an amplifier (5073PR, Olympus Corporation, Tokyo, Japan). Both transducers were acoustically coupled to the bone sample using a transparent ultrasonic coupling agent. Concurrently, a 1 MHz focusing transducer (Olympus Corporation, Tokyo, Japan) was employed to receive the PA signal generated by the blackbody. Data acquisition was performed using a digital oscilloscope (HDO6000, Teledyne Lecroy, United States) set at a sampling rate of 250 MHz, which was deemed sufficient for our experimental requirements. Due to the anisotropic properties of cancellous bone, we employed a translation stage to move the bone sample longitudinally in 2 mm increments, for a total of three. This enabled us to capture PA signals at four distinct positions, thus providing a comprehensive evaluation of signal attenuation throughout the cancellous bone. To improve the signal-to-noise ratio, PA signals were recorded 50 times at each position and subsequently averaged.

### 5.4 PA Signal Processing

As shown in Fig. 5, the PA signal received by the hydrophone T1 closest to the light source serves as the unattenuated signal generated by the cancellous bone. Conversely, the PA signal received by transducer T2 located farther from the light source is a composite, reflecting both the inherent properties of the PA source and the acoustic propagation medium. By calculating the PA-DAS for both transducers, we can isolate and understand the frequency-related propagation characteristics specific to cancellous bone.

In the experiment, only longitudinal wave signals could be picked up because the gel was used as coupling agent. Moreover, the numerical results in Sec. 3.2 show that the attenuation of the slow P wave is much greater than that of the fast P wave, and the speed is much smaller than that of the fast P wave. Therefore, it is more meaningful to analyze the fast P wave signals with low attenuation than the complete signal.

**Fig. 6:**
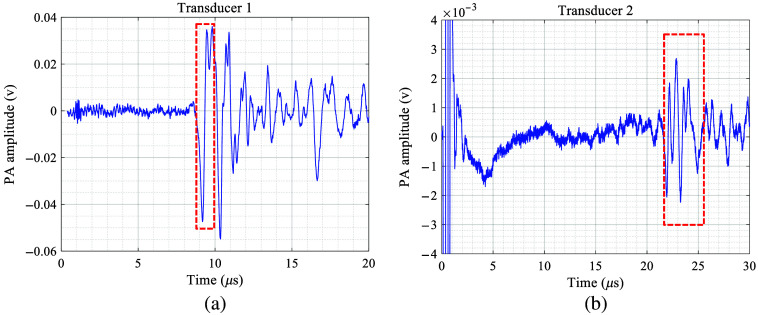
PA signals generated by bones received by (a) the transducer near the light source T1 and (b) the transducer away from the light source T2.

It is necessary to select a reasonable time window for more accurate spectral analysis. Thus we introduce the SoS for bone marrow $c_{\text{m}}=1500\;m/s$, which is larger than slow P wave and smaller than fast P wave, and bone trabecular $c_{\text{t}}=3200\;m/s$ (Ref. 66), which is larger than fast P wave, to calculate the start and end time of the picking window for fast-wave signals. As delineated by the red dotted box in Fig. 6(a), the direct PA signal duration for T1 is given by $t1=\frac{D_{\text{l}}}{c_{\text{m}}}=1.33\;\mu s$. The earliest and latest times for the PA signal of T2 were selected by $\frac{D_{\text{b}}-D_{\text{l}}}{c_{\text{t}}}$, and $\frac{D_{\text{b}}}{c_{\text{m}}}$, respectively. The length of the PA signal as captured by T2 is $t2=\frac{D_{\text{b}}}{c_{\text{m}}}-\frac{D_{\text{b}}-D_{\text{l}}}{c_{\text{t}}}=4.17\;\mu s$.

**Fig. 7:**
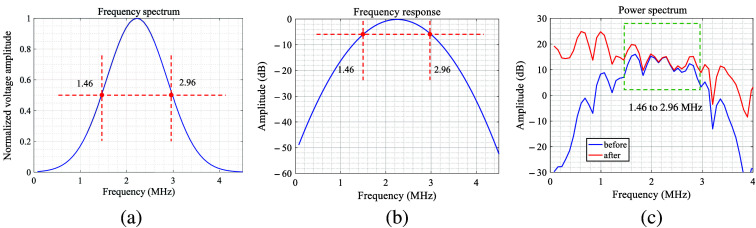
(a) Frequency–response curve of T2 provided by the Olympus Corporation (Tokyo, Japan). (a) Frequency–response curve of T2. Red intersection point denotes the frequency corresponding to the maximum value decreasing by 6 dB. (b) Spectrum of the PA signal of bone received by T2 (solid blue line) and spectrum after frequency–response correction (solid red line).

The frequency–response curve of the transducer T2, centered at 2.25 MHz, is presented in Fig. 7(a). As evident in this curve, the transducer produces its maximum output at the central frequency of 2.25 MHz and attenuates on either side, leading to distortion in the frequency domain of the PA signal as displayed by the oscilloscope. To more accurately investigate the spectral characteristics of the PA signal, it is necessary to correct the observed output signals in line with the frequency–response curve of the transducer. Corresponding to a 6 dB decline from the maximum frequency response to prevent excessive correction of high-frequency noise, a fitting frequency band ranging from 1.46 to 2.96 MHz was selected. After applying a 1 MHz high-pass filter to eliminate the influence of low-frequency noise, the spectral characteristics of the PA signal received by the distant transducer—both before and after minus the frequency response of transducer T2 for correction based on the frequency response provided by the Olympus Corporation (Tokyo, Japan)—are illustrated by the blue and red curves in Fig. 7(b). Although the frequency–response curve of the broadband hydrophone T1 is uniform, the signals received by T1 do not need correction. Given the data in Fig. 3, the linear fitting for the frequency curve was confined to the 1.46 to 2.96 MHz range.

To account for the spectral differences of the PA source caused by the anisotropy of cancellous bone, we calculated the PA-DAS As(f) based on Eq. (14) to evaluate the acoustic propagation characteristics of the bone:

$$\mathrm{As}(f)=10\;\mathrm{lg}(\frac{{\mathrm{PSD}}_{2}(f)}{{\mathrm{PSD}}_{1}(f)}).$$ (14)

**Fig. 8:**
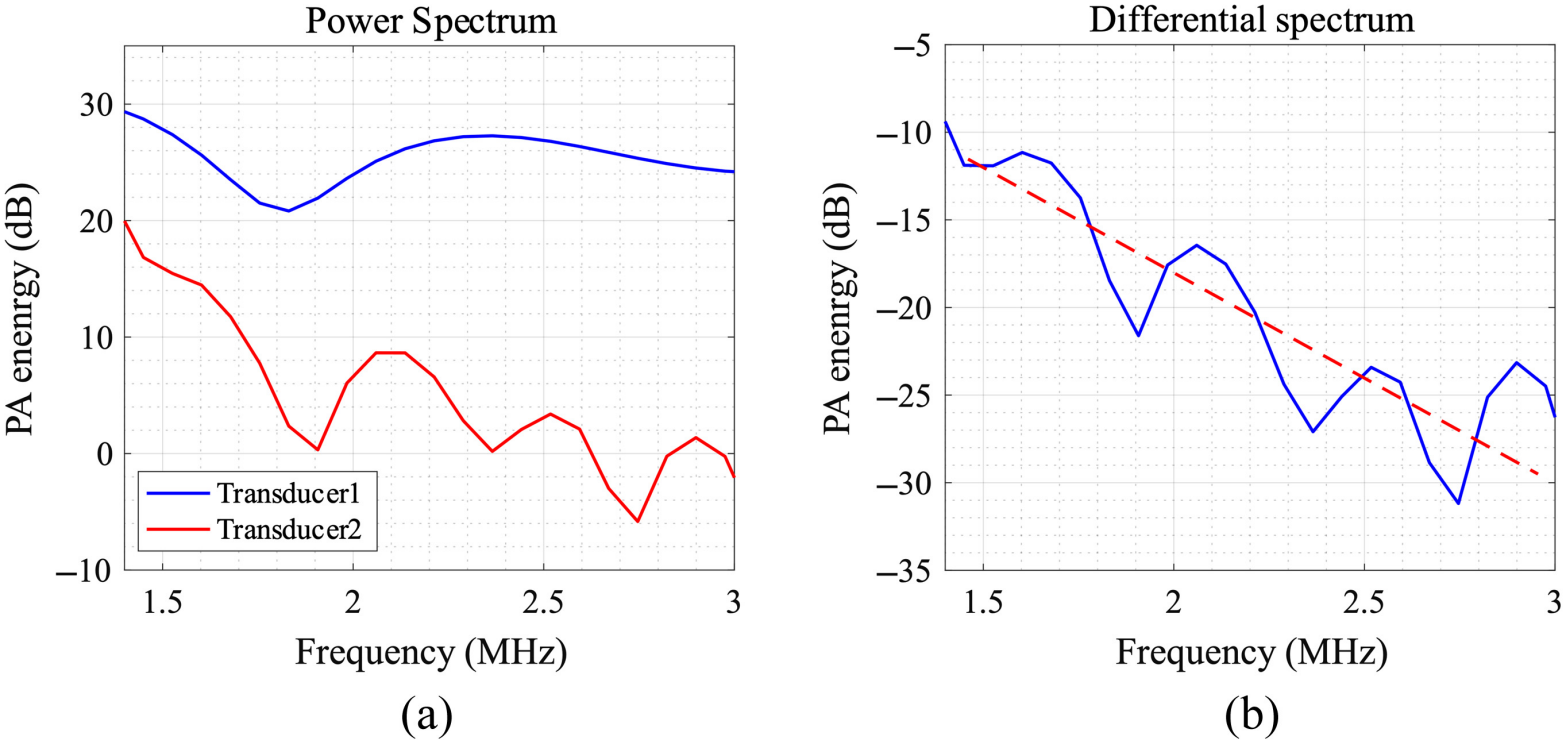
(a) Spectrum of PA signals received by the two transducers. (b) Differential spectrum of PA signal received by the two transducers.

Here ${\mathrm{PSD}}_{1}$ and ${\mathrm{PSD}}_{2}$ represent the power spectra of PA signals received by transducers 1 and 2, respectively, as depicted in Fig. 8(a). Notably, the PA signal from the transducer closer to the light source includes a higher concentration of high-frequency components due to the absence of propagation attenuation. In contrast, the PA signal from the transducer situated farther from the light source was mainly composed of low-frequency components owing to significant propagation attenuation in cancellous bone.

Linear fitting was performed for the PA-DAS in the frequency range of 1.46 to 2.96 MHz. From this, we extracted the absolute value of slope—slope—as a quantization parameter, enabling us to obtain the frequency-related attenuation of the PA signal as it travels through the bone.

### 5.5 Results

**Fig. 9:**
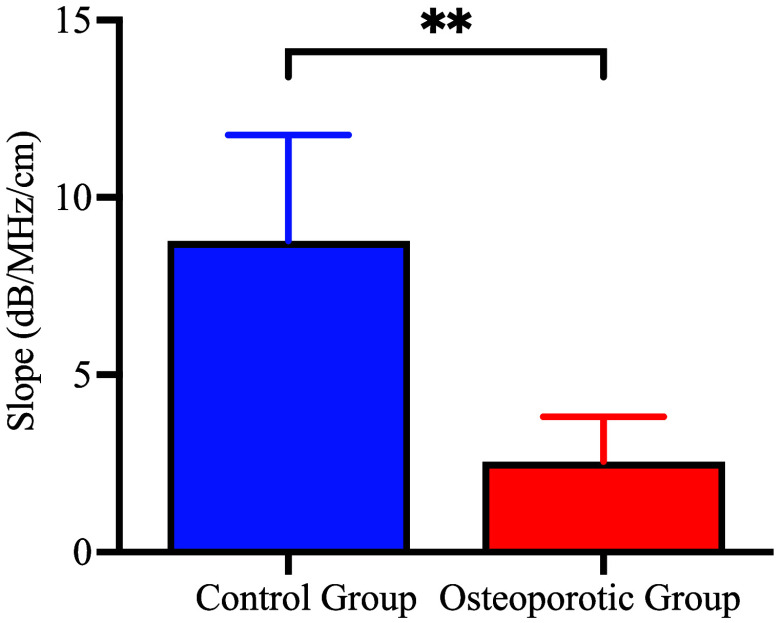
Statistical results of bone slope in the two studied groups (**p<0.01).

Figure 9 presents the slopes for both normal and osteoporotic bones, revealing a significantly lower slope for osteoporotic bones when compared with normal ones (**p<0.01). The average slope of the normal group is 8.77  dB/cm/MHz and the osteoporosis is 2.55  dB/cm/MHz. The attenuation coefficient of trabecular bone given in literature is 9.94  dB/cm/MHz.^83^ Ignoring the attenuation of bone marrow, the attenuation coefficients of cancellous bone with porosity ϕ can be calculated by 9.94×(1−ϕ)  dB/cm/MHz. It is the same order of magnitude as our experimental results but lower due to the ignorance of bone marrow attenuation and scattering attenuation, proving the rationality of our experiment design and implementation. Given that normal bone has higher BMD and lower porosity, the attenuation of high-frequency components is stronger during PA signal propagation through the cancellous bone structure. This results in a decreased high-to-low frequency ratio in the spectral distribution, leading to a larger slope for the control group. Conversely, osteoporotic bones have a slower slope, due to the weakening of the solid–liquid interface coupling and the decrease of viscosity attenuation caused by lower BMD and higher porosity. The experimental results qualitatively agree with the numerical simulation outcomes, quantitative comparisons are discussed in Sec. 6.

## 6 Conclusion and Discussion

PA technology holds immense promise for bone assessment and imaging. Researchers have proposed various spectral analysis methods in PA bone assessment, such as physicochemical^44^ and time-frequency spectrum,^84^^,^^85^ to discern characteristics of the bone microstructure and chemical composition of bone. However, achieving high-precision PAI to visualize the distribution of bone marrow clusters in the bone remains challenging due to the ultrawideband spectrum (from 220 kHz to 13.5 MHz in cancellous bone, see Appendix A2 in the Supplementary Material) and high frequency of PA signals generated by cancellous bone. Comprehending the attenuation of PA signals in cancellous bone is a pivotal cornerstone for high-resolution PAI of cancellous bone. The propagation of PA waves generated by bone marrow (mainly lipids) through the cancellous bone, a solid–liquid two-phase porous medium was studied theoretically, numerically, and experimentally in this study. Biot’s theory was modified to account for high frequencies and viscosity, providing an analytical solution for the broadband PA signal’s behavior in cancellous bone. Numerical simulation results show that when porosity is greater than 0.72, the viscous absorption attenuation of PA signals increases with frequency but decreases with greater porosity. Additionally, the fast P wave with high speed and low attenuation is more suitable for the detection of cancellous bone. In PA detection, to isolate the acoustic propagation properties of porous cancellous bone from the aliasing with the PA source, a PA-DAS method was proposed and a parameter slope was extracted to quantify frequency-specific acoustic attenuation. Experimental data on a rabbit osteoporotic model demonstrate that PA-DAS can effectively distinguish normal from osteoporotic bone, validating its utility for bone evaluation.

As shown in Fig. 9, the average slope of the normal group measured 8.77  dB/cm/MHz, and the osteoporosis group exhibited a slope of 2.55  dB/cm/MHz. Nevertheless, the slopes obtained from numerical simulations were 0.61  dB/cm/MHz for the normal bone and 0.19  dB/cm/MHz for the osteoporosis bone, as detailed in Appendix A1 in the Supplementary Material. Although the qualitative agreement between the experimental and simulated results is evident, quantitative disparities still exist. This is because cancellous bone’s acoustic propagation properties are predominantly governed by absorption and scattering of viscous solid–liquid two-phase porous structure.^86^ Absorption attenuation is an energy dissipation process caused by viscous friction at the fluid–solid interface, whereas scattering attenuation^87^ arises from reflections and scattering due to acoustic disparities between solid trabecular bone and liquid bone marrow, leading to the reduction of acoustic energy along the original propagation direction.^88^ This study focused on high-frequency viscous absorption attenuation in porous media, refining Biot’s theory to address higher frequency bands in PA signals and the viscosity of bone marrow. However, for more accurate quantification of scattering characteristics of cancellous bone, future studies should consider the anisotropy and heterogeneity intrinsic to its structure. Thankfully, the scattering attenuation in cancellous bone also adheres to a linear law with frequency.^87^^,^^89^ The proposed method PA-DAS in this paper can be readily extended due to this approximation.

When considering the clinical applications of PA-DAS, it is crucial to account for the layers of cortical bone and soft tissue that envelop cancellous bone. As the transducers are located on opposite sides of the bone, the thickness of the dense bone and soft tissue on both sides of the bone should be considered. Our previous studies successfully isolated PA signals originating from soft tissue, cortical bone, and cancellous bone in the time domain.^45^ Using the SoS to calculate the thickness of these three types of media and employing the exponential attenuation law to consider the optical and ultrasonic propagation attenuation of cortical bone and soft tissue, we can compensate the PA signals in further studies to isolate and analyze the pure PA signal corresponding to cancellous bone.^90^^–^^92^ In addition, the diameter of calcaneus bone in the human body is in the range of 2 to 3 cm.^43^ The attenuation of the cancellous bone to the high-frequency PA signal is stronger, and the received signal is mainly concentrated in the lower frequency band between 220 kHz and 1 MHz. According to the numerical simulation results shown in Fig. 3, the relationship between the attenuation coefficient and frequency is nonlinear at frequencies lower than 1 MHz. Therefore, it is necessary to fit the variation of low-frequency attenuation coefficient with frequency using a nonlinear function before applying this method to clinical practice.

## Supplementary Material







## Data Availability

The data that support the findings of this article are not publicly available due to privacy. The code can be requested from the author at q.cheng@tongji.edu.cn

## References

[1] Cummings S. R. Melton L. J., “Epidemiology and outcomes of osteoporotic fractures,” Lancet 359(9319), 1761–1767 (2002). LANCAO 0140-6736 10.1016/S0140-6736(02)08657-9 12049882

[2] Reginster J. Y. Burlet N., “Osteoporosis: a still increasing prevalence,” Bone 38(2 Suppl. 1), S4–S9 (2006). 10.1016/j.bone.2005.11.024 16455317

[r3] Jolly J. J. et al., “Protective effects of selected botanical agents on bone,” Int. J. Environ. Res. Public Health 15(5), 963 (2018). 10.3390/ijerph15050963 29751644 PMC5982002

[4] Cooper C. Campion G. Melton L. J., “Hip fractures in the elderly: a world-wide projection,” Osteoporosis Int. 2(6), 285–289 (1992). OSINEP 1433-2965 10.1007/BF01623184 1421796

[5] Wear K. A., “Mechanisms of interaction of ultrasound with cancellous bone: a review,” IEEE Trans. Ultrason. Ferroelectr. Freq. Control 67(3), 454–482 (2020). ITUCER 0885-3010 10.1109/TUFFC.2019.2947755 31634127 PMC7050438

[r6] Seeman E., “Age- and menopause-related bone loss compromise cortical and trabecular microstructure,” J. Gerontol. A Biol. Sci. Med. Sci. 68(10), 1218–1225 (2013). 10.1093/gerona/glt071 23833200

[r7] Vesterby A. Gundersen H. J. Melsen F., “Star volume of marrow space and trabeculae of the first lumbar vertebra: sampling efficiency and biological variation,” Bone 10(1), 7–13 (1989). 10.1016/8756-3282(89)90140-3 2660885

[8] Griffith J. F., “Age-related changes in the bone marrow,” Curr. Radiol. Rep. 5(6), 24 (2017). 10.1007/s40134-017-0218-8

[9] Pisani P. et al., “Screening and early diagnosis of osteoporosis through X-ray and ultrasound based techniques,” World J. Radiol. 5(11), 398–410 (2013). 10.4329/wjr.v5.i11.398 24349644 PMC3856332

[r10] Kanis J. A., “Assessment of fracture risk and its application to screening for postmenopausal osteoporosis: synopsis of a WHO report,” Osteoporosis Int. 4, 368–381 (1994). OSINEP 1433-2965 10.1007/BF01622200 7696835

[r11] Albanese C. V. De Terlizzi F. Passariello R., “Quantitative ultrasound of the phalanges and DXA of the lumbar spine and proximal femur in evaluating the risk of osteoporotic vertebral fracture in postmenopausal women,” Radiol. Med. 116(1), 92–101 (2011). 10.1007/s11547-010-0577-1 20927655

[12] Njeh C. F. Boivin C. M. Langton C. M., “The role of ultrasound in the assessment of osteoporosis: a review,” Osteoporosis Int. 7(1), 7–22 (1997). OSINEP 1433-2965 10.1007/BF01623454 9102067

[13] Cummings S. R. et al., “Risk factors for hip fracture in white women. Study of Osteoporotic Fractures Research Group,” N. Engl. J. Med. 332(12), 767–773 (1995). NEJMAG 0028-4793 10.1056/NEJM199503233321202 7862179

[14] Glüer C. C. et al., “Three quantitative ultrasound parameters reflect bone structure,” Calcif. Tissue Int. 55(1), 46–52 (1994). CTINDZ 0171-967X 10.1007/BF00310168 7922789

[15] Adams J. E., “Quantitative computed tomography,” Eur. J. Radiol. 71(3), 415–424 (2009). EJRADR 0720-048X 10.1016/j.ejrad.2009.04.074 19682815

[16] Liu X. S. et al., “Bone density, geometry, microstructure, and stiffness: relationships between peripheral and central skeletal sites assessed by DXA, HR-pQCT, and cQCT in premenopausal women,” J. Bone Miner. Res. 25(10), 2229–2238 (2010). JBMREJ 0884-0431 10.1002/jbmr.111 20499344 PMC3128822

[17] Gee C. S. et al., “Validation of bone marrow fat quantification in the presence of trabecular bone using MRI,” J. Magn. Reson. Imaging 42(2), 539–544 (2015). 10.1002/jmri.24795 25425074 PMC4442769

[r18] Yu H. et al., “Multiecho reconstruction for simultaneous water-fat decomposition and $T_{2}*$ estimation,” J. Magn. Reson. Imaging 26(4), 1153–1161 (2007). 10.1002/jmri.21090 17896369

[19] Yu H. et al., “Field map estimation with a region growing scheme for iterative 3-point water-fat decomposition,” Magn. Reson. Med. 54(4), 1032–1039 (2005). MRMEEN 0740-3194 10.1002/mrm.20654 16142718

[20] Griffith J. F. et al., “Vertebral bone mineral density, marrow perfusion, and fat content in healthy men and men with osteoporosis: dynamic contrast-enhanced MR imaging and MR spectroscopy,” Radiology 236(3), 945–951 (2005). RADLAX 0033-8419 10.1148/radiol.2363041425 16055699

[21] Griffith J. F. et al., “Vertebral marrow fat content and diffusion and perfusion indexes in women with varying bone density: MR evaluation,” Radiology 241(3), 831–838 (2006). RADLAX 0033-8419 10.1148/radiol.2413051858 17053202

[22] Chin K. Y. Ima-Nirwana S., “Calcaneal quantitative ultrasound as a determinant of bone health status: what properties of bone does it reflect?,” Int. J. Med. Sci. 10(12), 1778–1783 (2013). 10.7150/ijms.6765 24273451 PMC3837236

[23] Matsukawa M., “Bone ultrasound,” Jpn. J. Appl. Phys. 58(SG), SG0802 (2019). 10.7567/1347-4065/ab0dfa

[24] Grimal Q. Laugier P., “Quantitative ultrasound assessment of cortical bone properties beyond bone mineral density,” IRBM 40(1), 16–24 (2019). 10.1016/j.irbm.2018.10.006

[r25] Ta D. et al., “Measurement of the dispersion and attenuation of cylindrical ultrasonic guided waves in long bone,” Ultrasound Med. Biol. 35(4), 641–652 (2009). USMBA3 0301-5629 10.1016/j.ultrasmedbio.2008.10.007 19153000

[26] Grimal Q. et al., “Quantitative ultrasound of cortical bone in the femoral neck predicts femur strength: results of a pilot study,” J. Bone Miner. Res. 28(2), 302–312 (2013). JBMREJ 0884-0431 10.1002/jbmr.1742 22915370

[27] Kaufman J. J. Einhorn T. A., “Perspectives: ultrasound assessment of bone,” J. Bone Miner. Res. 8(5), 517–525 (1993). JBMREJ 0884-0431 10.1002/jbmr.5650080502 8511979

[28] Huang S. et al., “Interstitial assessment of aggressive prostate cancer by physio-chemical photoacoustics: an ex vivo study with intact human prostates,” Med. Phys. 45, 4125–4132 (2018). MPHYA6 0094-2405 10.1002/mp.13061 PMC6629517 29935081

[r29] Mallidi S. et al., “Prediction of tumor recurrence and therapy monitoring using ultrasound-guided photoacoustic imaging,” Theranostics 5(3), 289–301 (2015). 10.7150/thno.10155 25553116 PMC4279192

[r30] Cao R. et al., “Functional and oxygen-metabolic photoacoustic microscopy of the awake mouse brain,” NeuroImage 150, 77–87 (2017). NEIMEF 1053-8119 10.1016/j.neuroimage.2017.01.049 28111187 PMC5391672

[r31] Oh J. T. et al., “Three-dimensional imaging of skin melanoma in vivo by dual-wavelength photoacoustic microscopy,” J. Biomed. Opt. 11(3), 034032 (2006). JBOPFO 1083-3668 10.1117/1.2210907 16822081

[32] Xiang L. Z. Zhou F. F., “Photoacoustic imaging application in tumor diagnosis and treatment monitoring,” Key Eng. Mater. 364–366, 1100–1104 (2007). 10.4028/www.scientific.net/KEM.364-366.1100

[33] Lashkari B. Mandelis A., “Coregistered photoacoustic and ultrasonic signatures of early bone density variations,” J. Biomed. Opt. 19(3), 036015 (2014). JBOPFO 1083-3668 10.1117/1.JBO.19.3.036015 24647973

[34] Yang L. et al., “Photoacoustic and ultrasound imaging of cancellous bone tissue,” J. Biomed. Opt. 20(7), 076016 (2015). JBOPFO 1083-3668 10.1117/1.JBO.20.7.076016 26222963

[r35] Yang L. et al., “Bone composition diagnostics: photoacoustics versus ultrasound,” Int. J. Thermophys. 36(5-6), 862–867 (2015). IJTHDY 0195-928X 10.1007/s10765-014-1701-6

[36] Lashkari B. Yang L. Mandelis A., “The application of backscattered ultrasound and photoacoustic signals for assessment of bone collagen and mineral contents,” Quantum Imaging Med. Surg. 5(1), 46–56 (2015). 10.3978/j.issn.2223-4292.2014.11.11 PMC4312292 25694953

[37] Wang X. et al., “Photoacoustic measurement of bone health: a study for clinical feasibility,” in IEEE Int. Ultrasonics Symp. (IUS), IEEE, Tours, France, pp. 1–4 (2016). 10.1109/ULTSYM.2016.7728418

[r38] Feng T. et al., “Characterization of bone microstructure using photoacoustic spectrum analysis,” Proc. SPIE 9323, 93234I (2015). PSISDG 0277-786X 10.1117/12.2078258 PMC4646513 26406719

[39] Feng T. et al., “Bone assessment via thermal photo-acoustic measurements,” Opt. Lett. 40(8), 1721–1724 (2015). OPLEDP 0146-9592 10.1364/OL.40.001721 25872057 PMC4470252

[40] Feng T. et al., “Functional photoacoustic and ultrasonic assessment of osteoporosis: a clinical feasibility study,” BME Front. 2020, 1081540 (2020). 10.34133/2020/1081540 37849970 PMC10521673

[41] Steinberg I. et al., “First-in-human study of bone pathologies using low-cost and compact dual-wavelength photoacoustic system,” IEEE J. Select. Top. Quantum Electron. 25(1), 1–8 (2019). IJSQEN 1077-260X 10.1109/JSTQE.2018.2866702

[42] Weiss M. et al., “Reference database for bone speed of sound measurement by a novel quantitative multi-site ultrasound device,” Osteoporos. Int. 11(8), 688–696 (2000). OSINEP 1433-2965 10.1007/s001980070067 11095172

[43] Feng T. et al., “The feasibility study of the transmission mode photoacoustic measurement of human calcaneus bone in vivo,” Photoacoustics 23, 100273 (2021). 10.1016/j.pacs.2021.100273 34745881 PMC8552339

[44] Feng T. et al., “Characterization of multi-biomarkers for bone health assessment based on photoacoustic physicochemical analysis method,” Photoacoustics 25, 100320 (2022). 10.1016/j.pacs.2021.100320 35004172 PMC8717597

[45] Feng T. et al., “Feasibility study for bone health assessment based on photoacoustic imaging method,” Chin. Opt. Lett. 18(12), 121704 (2020). CJOEE3 1671-7694

[46] Xie W. et al., “Photoacoustic characterization of bone physico-chemical information,” Biomed. Opt. Express 13(5), 2668–2681 (2022). BOEICL 2156-7085 10.1364/BOE.457278 35774314 PMC9203098

[47] Xie W. et al., “Bone microstructure evaluation by photoacoustic time-frequency spectral analysis,” in IEEE Int. Ultrasonics Symp. (IUS), IEEE, Las Vegas, Nevada, pp. 1–4 (2020). 10.1109/IUS46767.2020.9251672

[48] Gonzalez E. A. Bell M. A. L., “Photoacoustic imaging and characterization of bone in medicine: overview, applications, and outlook,” Annu. Rev. Biomed. Eng. 25, 207–232 (2023). ARBEF7 1523-9829 10.1146/annurev-bioeng-081622-025405 37000966

[49] Fellah Z. E. A. et al., “Ultrasonic wave propagation in human cancellous bone: application of Biot theory,” J. Acoust. Soc. Am. 116(1), 61–73 (2004). JASMAN 0001-4966 10.1121/1.1755239 15295965

[50] Biot M. A., “General theory of three‐dimensional consolidation,” J. Appl. Phys. 12(2), 155–164 (1941). JAPIAU 0021-8979 10.1063/1.1712886

[r51] Biot M. A., “Theory of elasticity and consolidation for a porous anisotropic solid,” J. Appl. Phys. 26(2), 182–185 (1955). JAPIAU 0021-8979 10.1063/1.1721956

[r52] Biot M. A., “Theory of deformation of a porous viscoelastic anisotropic solid,” J. Appl. Phys. 27(5), 459–467 (1956). JAPIAU 0021-8979 10.1063/1.1722402

[r53] Biot M. A. Willis D. G., “The elastic coefficients of the theory of consolidation,” J. Appl. Mech. 24(4), 594–601 (1957). JAMCAV 0021-8936 10.1115/1.4011606

[r54] Biot M. A., “Theory of propagation of elastic waves in a fluid‐saturated porous solid. II. Higher frequency range,” J. Acoust. Soc. Am. 28(2), 179–191 (1956). JASMAN 0001-4966 10.1121/1.1908241

[55] Biot M. A., “Theory of propagation of elastic waves in a fluid‐saturated porous solid. I. Low‐frequency range,” J. Acoust. Soc. Am. 28(2), 168–178 (1956). JASMAN 0001-4966 10.1121/1.1908239

[56] Fry F. J. Barger J. E., “Acoustical properties of the human skull,” J. Acoust. Soc. Am. 63(5), 1576–1590 (1978). JASMAN 0001-4966 10.1121/1.381852 690336

[r57] Ashman R. B. Corin J. D. Turner C. H., “Elastic properties of cancellous bone: measurement by an ultrasonic technique,” J. Biomech. 20(10), 979–986 (1987). JBMCB5 0021-9290 10.1016/0021-9290(87)90327-7 3693379

[r58] Fellah Z. A. et al., “Application of the Biot model to ultrasound in bone: direct problem,” IEEE Trans. Ultrason. Ferroelectr. Freq. Control 55(7), 1508–1515 (2008). ITUCER 0885-3010 10.1109/TUFFC.2008.826 18986940

[r59] Sebaa N. et al., “Application of the Biot model to ultrasound in bone: inverse problem,” IEEE Trans. Ultrason. Ferroelectr. Freq. Control 55(7), 1516–1523 (2008). ITUCER 0885-3010 10.1109/TUFFC.2008.827 18986941

[r60] Sadouki M. et al., “Ultrasonic propagation of reflected waves in cancellous bone: application of Biot theory,” in 6th Eur. Symp. Ultrasonic Characterization of Bone, IEEE, Corfu, Greece, pp. 1–4 (2015). 10.1109/ESUCB.2015.7169900

[r61] Pakula M. et al., “Application of Biot’s theory to ultrasonic characterization of human cancellous bones: determination of structural, material, and mechanical properties,” J. Acoust. Soc. Am. 123(4), 2415–2423 (2008). JASMAN 0001-4966 10.1121/1.2839016 18397044

[62] Hodaei M. Maghoul P. Wu N., “Three-dimensional biomechanical modeling of cylindrical bone-like porous materials subject to acoustic waves,” Int. J. Mech. Sci. 213, 106835 (2022). IMSCAW 0020-7403 10.1016/j.ijmecsci.2021.106835

[63] Wyllie M. R. J. Gardner G. H. F. Gregory A. R., “Studies of elastic wave attenuation in porous media,” Geophysics 27(5), 569–589 (1962). GPYSA7 0016-8033 10.1190/1.1439063

[64] Hughes E. R. et al., “Ultrasonic propagation in cancellous bone: a new stratified model,” Ultrasound Med. Biol. 25(5), 811–821 (1999). USMBA3 0301-5629 10.1016/S0301-5629(99)00034-4 10414898

[65] Hughes E. R. et al., “Estimation of critical and viscous frequencies for Biot theory in cancellous bone,” Ultrasonics 41(5), 365–368 (2003). ULTRA3 0041-624X 10.1016/S0041-624X(03)00107-0 12788218

[66] Bennamane A. Boutkedjirt T., “Theoretical and experimental study of the ultrasonic attenuation in bovine cancellous bone,” Appl. Acoust. 115, 50–60 (2017). AACOBL 0003-682X 10.1016/j.apacoust.2016.08.011

[67] Cox T. et al., “k-space propagation models for acoustically heterogeneous media: application to biomedical photoacoustics,” J. Acoust. Soc. Am. 121(6), 3453–3464 (2007). JASMAN 0001-4966 10.1121/1.2717409 17552697

[68] Sigrist M. W. Kneubühl F. K., “Laser‐generated stress waves in liquids,” J. Acoust. Soc. Am. 64(6), 1652–1663 (1978). JASMAN 0001-4966 10.1121/1.382132

[69] Johnson D. L. Koplik J. Dashen R., “Theory of dynamic permeability and tortuosity in fluid-saturated porous media,” J. Fluid Mech. 176, 379–402 (1987). JFLSA7 0022-1120 10.1017/S0022112087000727

[70] Feng T. et al., “Photoacoustic bone characterization: a progress review,” Chin. Sci. Bull. 68(26), 3437–3454 (2023). CSBUEF 1001-6538 10.1360/TB-2023-0335

[71] Hosokawa A. Otani T., “Ultrasonic wave propagation in bovine cancellous bone,” J. Acoust. Soc. Am. 101(1), 558–562 (1997). JASMAN 0001-4966 10.1121/1.418118 9000743

[72] McKelvie M. L. Palmer S. B., “The interaction of ultrasound with cancellous bone,” Phys. Med. Biol. 36(10), 1331–1340 (1991). PHMBA7 0031-9155 10.1088/0031-9155/36/10/003 1745661

[73] Cardoso L. et al., “In vitro acoustic waves propagation in human and bovine cancellous bone,” J. Bone Miner. Res. 18, 1803–1812 (2003). JBMREJ 0884-0431 10.1359/jbmr.2003.18.10.1803 14584891

[r74] Porrelli D. et al., “Trabecular bone porosity and pore size distribution in osteoporotic patients—a low field nuclear magnetic resonance and microcomputed tomography investigation,” J. Mech. Behav. Biomed. Mater. 125, 104933 (2022). 10.1016/j.jmbbm.2021.104933 34837800

[r75] Li B. et al., “Characterization of a rabbit osteoporosis model induced by ovariectomy and glucocorticoid,” Acta Orthop. 81(3), 396–401 (2010). 10.3109/17453674.2010.483986 20446884 PMC2876847

[r76] Ebaerhardt A.W. Jones A.Y. Blair H. C., “Regional trabecular bone matrix degeneration and osteocyte death in femora of glucocorticoid treated rabbits,” Endocrinology 142(3), 1333–1340 (2001). ENDOAO 0013-7227 10.1210/endo.142.3.8048 11181552

[77] Hodaei M. Mandelis A., “Quantitative osteoporosis diagnosis of porous cancellous bone using poroelastodynamic modal analysis,” J. Acoust. Soc. Am., 154, 3101–3124 (2023). JASMAN 0001-4966 10.1121/10.0022351 37966333

[78] Yao D.-K. et al., “Photoacoustic measurement of the Grüneisen parameter of tissue,” J. Biomed. Opt. 19(1), 017007 (2014). JBOPFO 1083-3668 10.1117/1.JBO.19.1.017007 24474512 PMC3904038

[79] Feng T. et al., “Detection of collagen by multi-wavelength photoacoustic analysis as a biomarker for bone health assessment,” Photoacoustics 24, 100296 (2021). 10.1016/j.pacs.2021.100296 34522607 PMC8426564

[80] Guglielmi G. Griffith J. F., “Bone marrow changes in osteoporosis,” in Osteoporosis and Bone Densitometry Measurements, Guglielmi G., Ed., pp. 69–85, Springer, Heidelberg, Berlin (2013)

[81] Wang H. W. et al., “Label-free bond-selective imaging by listening to vibrationally excited molecules,” Phys. Rev. Lett. 106(23), 238106 (2011). PRLTAO 0031-9007 10.1103/PhysRevLett.106.238106 21770549 PMC3398792

[82] Lei H. et al., “Characterizing intestinal inflammation and fibrosis in Crohn’s disease by photoacoustic imaging: feasibility study,” Biomed. Opt. Express 7(7), 2837–2848 (2016). BOEICL 2156-7085 10.1364/BOE.7.002837 27446710 PMC4948634

[83] Culjat M. O. et al., “A review of tissue substitutes for ultrasound imaging,” Ultrasound Med. Biol. 36(6), 861–873 (2019). USMBA3 0301-5629 10.1016/j.ultrasmedbio.2010.02.012 20510184

[84] Xie W. et al., “Wavelet transform-based photoacoustic time-frequency spectral analysis for bone assessment,” Photoacoustics 22, 100259 (2021). 10.1016/j.pacs.2021.100259 33777692 PMC7985564

[85] Xu W. Xie W. Cheng Q., “Intelligent photoacoustic diagnosis of osteoporosis based on wavelet scattering network,” in IEEE Int. Ultrasonics Symp. (IUS), IEEE, Montreal, Québec, pp. 1–4 (2023). 10.1109/IUS51837.2023.10306876

[86] Laugier P., “Quantitative ultrasound of bone: looking ahead,” Joint Bone Spine 73(2), 125–128 (2006). 10.1016/j.jbspin.2005.10.012 16488646

[87] Strelitzki R. Nicholson P. H. F. Paech V., “A model for ultrasonic scattering in cancellous bone based on velocity fluctuations in a binary mixture,” Physiol. Meas. 19(2), 189–196 (1998). PMEAE3 0967-3334 10.1088/0967-3334/19/2/006 9626683

[88] Insana M. F. et al., “Describing small‐scale structure in random media using pulse‐echo ultrasound,” J. Acoust. Soc. Am. 87(1), 179–192 (1990). JASMAN 0001-4966 10.1121/1.399283 2299033 PMC2745727

[89] Sehgal C. M. Greenleaf J. F., “Scattering of ultrasound by tissues,” Ultrasonic Imaging 6(1), 60–80 (1984). ULIMD4 0161-7346 10.1177/016173468400600106 6540912

[90] Pifferi A. et al., “Optical biopsy of bone tissue: a step toward the diagnosis of bone pathologies,” J. Biomed. Opt. 9(3), 474–480 (2004). JBOPFO 1083-3668 10.1117/1.1691029 15189084

[r91] Liu C. et al., “The analysis and compensation of cortical thickness effect on ultrasonic backscatter signals in cancellous bone,” J. Appl. Phys. 116(12), 124903 (2014). JAPIAU 0021-8979 10.1063/1.4896258

[92] Firbank M. et al., “Measurement of the optical properties of the skull in the wavelength range 650-950 nm,” Phys. Med. Biol. 38(4), 503–510 (1993). PHMBA7 0031-9155 10.1088/0031-9155/38/4/002 8488176

