# Supplementary material for: Theoretical and experimental study of attenuation in cancellous bone
Source: J Biomed Opt. 2024 Mar 19;29(Suppl 1):S11526. doi: 10.1117/1.JBO.29.S1.S11526 (PMC10949015; doi:10.1117/1.JBO.29.S1.S11526)
Supplement: Supplementary file 1 [file JBO_029_S11526_SD001.pdf]

Appendix A: Supplemental Material

A.1 The slope of numerical simulation attenuation coefficients

To better compare the numerical simulation with the experimental results, we linearly fit the fast wave attenuation coefficients obtained by numerical simulation in the frequency band 1.46-2.96 MHz, as shown in Fig. S1. The slopes of cancellous bone with different porosities were obtained, as shown in Table S1.

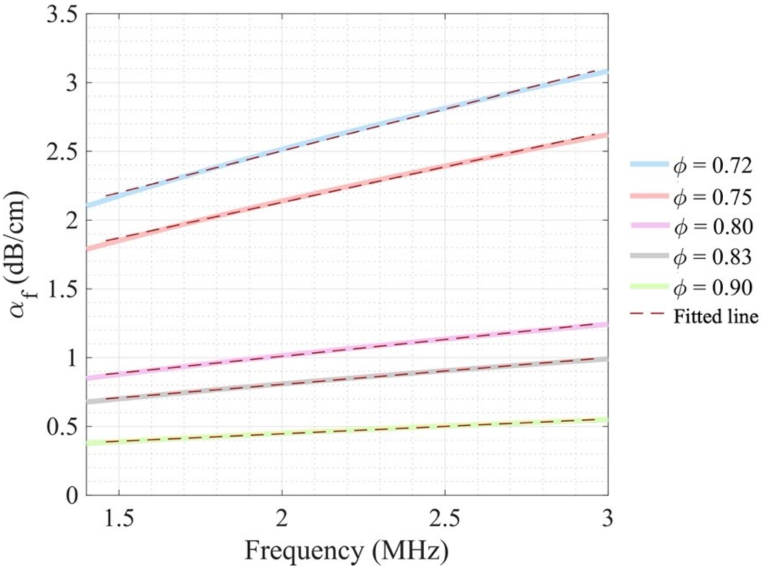

Fig. S1 Linear fit of the fast wave attenuation coefficients obtained by numerical simulation in the frequency band of 1.46–2.96 MHz.

Table S1 Slopes estimated based on the linear fittings of fast wave attenuation coefficients obtained by numerical simulations in the frequency band of 1.46–2.96 MHz.

**Porosity**	0.72	0.75	0.80	0.83	0.90
**Slope (dB/cm/MHz)**	0.61	0.52	0.24	0.19	0.11

Based on the results of Micro-CT shown in Fig. 4d, the numerical simulation results with the porosity of 0.72 were selected as the representative of the normal group, and the numerical simulation results with the porosity of 0.83 represented the

osteoporosis group. In other words, in the numerical simulation results, the slope of the normal group is 0.61 dB/cm/MHz, and the slope of the osteoporosis group is 0.19 dB/cm/MHz.

A.2 Frequency of PA signals generated by cancellous bone

The symbol f_c in the manuscript represents the medium's critical frequency, approximately 1-10 kHz in cancellous bone[64,65]. Sigrist and Kneubühl[68] proposed the solution of the PA field of a spherical source, and the sound pressure of position $\boldsymbol{r}$ at time t is given by,

$$p(\boldsymbol{r},t) = -\frac{\beta E_a c^2}{2\pi^{\frac{3}{2}}\sigma r_0^3}\frac{1}{r}(t-r)\exp\left\{-\left[\frac{c}{r_0}\left(t-\frac{r}{c}\right)\right]^2\right\} \quad \text{(S1)}$$

The frequency spectrum of the PA field induced by the spherical source can be obtained by the Fourier transform:

$$P(r,\omega) = j\frac{\beta E_a C^2}{2\pi\sigma r_0^2}\frac{1}{r}\omega e^{-\frac{\omega^2}{4}} \quad \text{(S2)}$$

where, ω represents the equivalent angular frequency. The relationship between ω and the angular frequency ω_0 of PA signal generated by the source is $\omega_0 = \frac{c}{r_0}\omega$. When the first-order derivative of the spectrum with respect to ω equals zero, the spectrum exhibits a maximum. At this point, the equivalent angular frequency $\omega = \sqrt{2}$, and the angular frequency of the PA signal generated by the spherical source is given by $\omega_0 = \sqrt{2}\frac{c}{r_0}$, and the frequency is $f = \frac{\omega_0}{2\pi}$.

As shown, the larger the target size, the lower the frequency of the generated PA signal. As we mentioned in the manuscript, the sizes of trabecular bone vary from 50 to 200 μm, while the trabecular spaces (bone marrow clusters) range from 0.2 to 3 mm[66].

The lowest frequency of PA signal is generated when the size of the bone marrow cluster is 3 mm (i.e., $r_0 = 1.5\,mm$), approximately 220 kHz. The highest frequency of PA signal generated by bone marrow cluster is approximately 3.4 MHz ($r_0 = 0.1\,mm$). The highest frequency of PA signal is generated when trabecular bone thickness is 50 μm (i.e., $r_0 = 25\,\mu m$), approximately 13.5 MHz. Therefore, the frequency range of the PA signal generated by cancellous bone is much higher than that of f_c.

A.3 Energy Calibration

We used 10% laser energy irradiating a blackbody to calibrate the result by getting rid of the oscillation of laser energy. The laser energy at the same wavelength has vibrated over time. As shown in Fig. 5 in our manuscript, through the beam splitter, 90% of the laser energy is irradiated on the sample to stimulate the photoacoustic signal, and 10% of the energy is irradiated on the blackbody on the wall of the water tank. The transducer receives the photoacoustic signal generated by the black body through water coupling in the water tank. Due to the blackbody being stable and having a flat optical absorption spectrum, the factor that affects the blackbody signal amplitude is the laser energy. We choose the peak-to-peak value of the blackbody signal of a certain experiment as the benchmark and calibrate the signals obtained from multiple experiments of different samples and sampling points, eliminating the impact of laser energy oscillation with time.
